# Maternal and paternal involvement in complementary feeding in Kaduna State, Nigeria: The continuum of gender roles in urban and rural settings

**DOI:** 10.1111/mcn.13325

**Published:** 2022-01-25

**Authors:** Diana Allotey, Valerie L. Flax, Abiodun Ipadeola, Sarah Kwasu, Margaret E. Bentley, Beamlak Worku, Keerti Kalluru, Carmina G. Valle, Sujata Bose, Stephanie L. Martin

**Affiliations:** ^1^ Department of Nutrition, Gillings School of Global Public Health University of North Carolina at Chapel Hill Chapel Hill North Carolina USA; ^2^ RTI International Research Triangle Park North Carolina USA; ^3^ Datametrics Associates Limited Abuja Nigeria; ^4^ Alive & Thrive Kaduna Kaduna State Nigeria; ^5^ Alive & Thrive Washington District of Columbia USA

**Keywords:** caregivers, child, fathers, focus groups, gender role, mothers, Nigeria

## Abstract

Household gender roles influence infant and young child feeding behaviours and may contribute to suboptimal complementary feeding practices through inequitable household decision‐making, intra‐household food allocation and limited paternal support for resources and caregiving. In Igabi local government area of Kaduna State, Nigeria, the Alive & Thrive (A&T) initiative implemented an intervention to improve complementary feeding practices through father engagement. This study describes household gender roles among A&T participants and how they influence maternal and paternal involvement in complementary feeding. We conducted 16 focus group discussions with mothers and fathers of children aged 6–23 months in urban and rural administrative wards and analysed them using qualitative thematic analysis methods. Most mothers and fathers have traditional roles with fathers as ‘providers’ and ‘supervisors’ and mothers as ‘caregivers’. Traditional normative roles of fathers limit their involvement in ‘hands‐on’ activities, which support feeding and caring for children. Less traditional normative roles, whereby some mothers contributed to the provision of resources and some fathers contributed to caregiving, were also described by some participants and were more salient in the urban wards. In the rural wards, more fathers expressed resistance to fathers playing less traditional roles. Fathers who participated in caregiving tasks reported respect from their children, strong family relationships and had healthy home environments. Our research findings point to the need for more context‐specific approaches that address prevalent gender normative roles in complementary feeding in a variety of settings.

## Key messages

• Some mothers and fathers still hold traditional attitudes about their roles in feeding and caring for children.
• Traditional gender normative roles of fathers limit their involvement in the ‘hands‐on’ activities that support feeding and caring for children.
• Some mothers and fathers are performing ‘non‐traditional’ gender normative roles despite social disapproval.
• The shifts from the traditional roles of mothers and fathers are more salient in urban compared with rural wards.

## 1 INTRODUCTION

Optimal infant and young child feeding practices, including adequate safe and diverse complementary foods for children aged 6–23 months, have profound effects on child health and development (Bhutta et al., 2013; WHO, 2010). In addition to their importance for the growth and development of young children, appropriate complementary feeding with continued breastfeeding could prevent 6% of all under‐five deaths (Bhutta et al., 2008; Jones et al., 2003). Despite the health and growth benefits, estimates from 2010 to 2016 show that less than 20% of children aged 6–23 months in many countries in sub‐Saharan Africa are fed diets that meet the minimally acceptable standards for appropriate complementary feeding (Gebremedhin, 2019). In Nigeria, the most populous country in sub‐Saharan Africa, only 11% of children received a minimally acceptable diet in 2018 (National Population Commission and ICF, 2018). Nigeria thus contributes considerably to the burden of suboptimal complementary feeding practices in sub‐Saharan Africa.

Complementary feeding practices are influenced by food accessibility, including food availability, affordability and acceptability, and by the care and hygiene practices of caregivers (UNICEF, 2020). These are in turn driven by caregivers' knowledge and time, household dynamics and social norms, of which household gender norms and roles occupy a central position (UNICEF, 2020). In many communities in sub‐Saharan Africa, there are cultural norms that specify gender‐based roles and responsibilities in child health, nutrition and care (Dickin et al., 2021; Doyle et al., 2018). A study in Nigeria on the roles of mothers and fathers in child feeding and caregiving reported that men consider women to be solely responsible for feeding the family (Ene‐Obong et al., 2017). Another study in Malawi also reported that fathers consider anything related to caring for children, including feeding of children, as ‘awkward’ and a threat to ‘their masculinity’, speaking to the influence of gender‐related social norms in households with regard to feeding and caring for children (Bezner Kerr, 2016).

Gender‐related social norms include injunctive and descriptive household gender norms. Injunctive household gender norms represent perceptions of ‘what ought to be done’ (Cialdini et al., 1991; Cislaghi & Heise, 2019). They include perceptions of culturally acceptable behaviours of mothers and fathers in their homes relative to a gender‐based division of roles and responsibilities. Descriptive household gender norms are the perceptions of ‘what most others are doing’ (Cialdini et al., 1991; Cislaghi & Heise, 2019).

Findings from reviews (Dickin et al., 2021; Muralidharan et al., 2015; Schriver et al., 2017) and studies (Bégin et al., 1999; Chintalapudi et al., 2018; Engebretsen et al., 2010; Kulkarni et al., 2020; Shroff et al., 2011) suggest that pervasive traditional household gender norms and roles can negatively impact child health, nutrition and care through inequitable household decision‐making, intra‐household food allocation and limited paternal support in the provision of resources and caregiving. However, with the exception of a few studies in Northern Malawi and rural Zambia, there is limited intervention research that has examined the impact of household gender roles on complementary feeding (Bezner Kerr, 2005, 2016; Kumar et al., 2018). Additionally, to the best of our knowledge, none of the research on household gender roles and complementary feeding has compared urban and rural differences. This is an important consideration for developing context‐specific approaches that address prevailing injunctive and descriptive gender normative roles in complementary feeding in a variety of settings. This study set in Nigeria addresses these gaps by identifying and thematically describing how household gender roles influence complementary feeding, highlighting the roles mothers and fathers play in their homes and how these facilitate or inhibit feeding and caring for children. We also compare how these roles are different in urban and rural wards.

## 2 METHODS

### 2.1 Study context

Kaduna State is located in North Central Nigeria. The region is a mix of people of diverse ethnicities and religions, with the Hausas and Fulanis being the two predominant ethnic groups, while Islam and Christianity are the dominant religions (Adegboye et al., 2016; Angerbrandt, 2011; Wapwera & Gajere, 2017). The ethnoreligious groups in this region of Nigeria are largely patriarchal with fathers designated as the ‘leaders and ultimate decision makers’ within their families (Oguntunde et al., 2019; Sinai et al., 2017).

### 2.2 Design and sample

In September 2020, eight focus group discussions (FGDs) with mothers and eight FGDs with fathers (160 total mothers and fathers) of children aged 6–23 months were conducted as part of an evaluation of a 12‐month intervention to improve child complementary feeding outcomes through father engagement. Our objectives were to identify and thematically describe how household gender roles influence complementary feeding within the context of a father‐focused complementary feeding intervention.

The study was conducted in six administrative wards of the Igabi local government area of Kaduna State, Nigeria. Wards are the smallest of the three levels of administrative units (state, local government area and ward) in Nigeria (National Population Commission and ICF, 2018). The wards were purposefully selected to be representative of the mixture of urban and rural settings and ethnic and religious diversity, as well as the social setting (i.e. presence of CBOs), of Kaduna State. Out of the six wards selected, two wards were urban and four were rural. The rural wards included Zangon Aya, Igabi, Turunku and Kwarau, while the urban wards included Rigasa and Rigachikun. All the wards selected had been exposed to the intervention.

**Figure 1:**
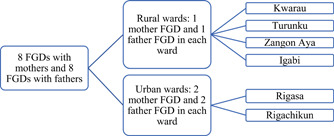
Locations of the focus group discussions with fathers and mothers

Mothers and fathers were eligible if they had a biological child 6–23 months of age, were 18 years or older and were cohabiting regardless of their marital status. Mothers were also eligible if they were 15–17 years of age, married and cohabiting with their husbands because Nigerian law considered such mothers as consenting adults. The sampling process involved, in consultation with community leaders, geographically dividing each of the six wards into three sections. A purposive sample of four eligible mothers and four eligible fathers who were willing to participate from each of the three sections were recruited. Out of 12 mothers and 12 fathers recruited, 10 participated in each mother and father FGD in each ward with the remaining two as backup participants. Although cohabitation with partner/spouse was part of the eligibility criteria for the mothers and fathers, mothers and fathers were not selected in pairs for the FGDs. Two FGDs each for mothers and fathers were conducted in each urban ward, and one FGD each for mothers and fathers occurred in each rural ward. A total of 16 FGDs were conducted with 8 FGDs each for mothers and fathers. All the mothers and fathers who were selected to participate in the FGDs had received the intervention Figure 1.

### 2.3 Intervention components

The intervention was part of the Alive & Thrive initiative in Nigeria and was implemented by the I Care Women and Youth Initiative (ICARE), a local Nigerian organization. As part of the implementation, ICARE trained 13 community‐based organizations (CBOs), 18 religious and traditional leaders (14 imams and 4 pastors) and 60 community health extension workers (CHEWs) and provided them with resources to carry out complementary feeding social and behaviour change communication (SBCC) activities. The resources included complementary feeding counselling cards for CBOs, talking points and sermon guides for religious and traditional leaders to educate fathers during meetings and religious services. The CHEWs were also provided with counselling cards and feeding bowls to use during home visits to counsel and reinforce complementary feeding messages with mothers and fathers (when present). CHEWs counselled mothers during home visits on feeding key food combinations designed to increase children's consumption of vitamin A‐rich vegetables and animal source foods and provided them with a feeding bowl that showed nutritious foods and the quantity of food to feed infants and children at different ages. In addition, complementary feeding SBCC messages were broadcast through radio and TV advertisements and mothers and fathers received leaflets with the same messages.

### 2.4 Data collection

After 12 months of intervention implementation, separate FGDs using specific discussion guides were conducted for mothers and fathers in rented halls. The FGDs were conducted in Hausa and English (where applicable) and moderated by Nigerian male and female research assistants and notetakers. All the research assistants and notetakers spoke Hausa, which is one of the major languages of the selected wards, lived in Kaduna State and were familiar with the terrain, culture and traditions of the people. They also had a college degree with extensive research experience (at least 5 years). They were also trained on the research study protocols and on qualitative research methodology.

The discussion guides included questions on the roles of mothers and fathers in the feeding and care of children, barriers and facilitators to child feeding and care experienced by mothers, and mothers' and fathers' perceptions of and experiences with fathers' involvement in child feeding and care. The fathers' FGD guide also included a vignette to elicit their perspectives on gender norms related to child feeding and care. Vignettes are short stories without an ending designed to be used in qualitative research to assess which combination of factors could influence beliefs or social norms (Hughes & Huby, 2012; Sivaram et al., 2004) and thus provided an opportunity to study descriptive and injunctive household gender normative roles. Vignettes have been used previously by other researchers in Nigeria to study power relations in contraceptive decision making (Adanikin et al., 2019).

The vignette for this study was adapted from a vignette used in a complementary feeding study in Tanzania (Martin et al., 2021). The vignette described two brothers, Yusuf and Kabiru, who had different levels of involvement (Kabiru was more involved than Yusuf) in the feeding and care of their children. The wife (Aisha) of the less‐involved father (Yusuf), in addition to providing and caring for the home and child, was struggling to feed their daughter (Laraba) diverse foods. Fathers were encouraged to reflect on the story, describe the current behaviours of the characters and suggest actions the characters might take, as well as possible endings to the story. Fathers were also asked to describe actions they take in their own homes related to the feeding and care of their children.

Each FGD lasted between 1 and 1.5 h. They were recorded using digital voice recorders, transcribed verbatim and translated into English, as needed by trained Nigerian transcriptionists. Ethical approval was obtained from the Kaduna State Ministry of Health and the institutional review board at RTI International. Participants provided written informed consent and received 2000 naira (approximately $5) or the equivalent in refreshments for their time.

### 2.5 Coding and analysis

Qualitative thematic analysis methods were used for coding and analysing the data from the FGDs. The analysis began with a thorough and repeated reading of all the transcripts to achieve ‘immersion and obtain a sense of the whole’ (Hsieh & Shannon, 2005; Tesch, 2013). Detailed thematic code books were then created for mothers and fathers using a combination of deductive and inductive codes (Fereday & Muir‐Cochrane, 2006).

Three coders used the qualitative data analysis software Atlas.ti (version 8) to code the transcripts. The coding process began with each coder independently coding one transcript from an FGD with mothers and one transcript from an FGD with fathers. The same two transcripts were coded by all the coders. The codes applied were compared and differences in code application were discussed to achieve consensus (Saldaña, 2009). Thereafter, one of the coders coded all the 16 transcripts and the other two coded 8 transcripts each. The double‐coded transcripts were then reviewed and discrepancies in code applications were discussed to achieve consensus.

Next, using Excel matrices of the relevant code queries, the spread of the codes was observed to identify patterns in the main themes across mothers' and fathers' groups and within urban and rural locations following the method described by Miles et al. (2014). The most salient themes were documented.

Furthermore, using follow‐up questions from the vignette included in the father FGDs about *the current behaviours of the characters (descriptive norms)* in contrast with *what the characters should do (injunctive norms)*, participants' perceptions of household gender norms regarding father involvement in feeding and caring for their children were described (Cialdini et al., 1991; Cislaghi & Heise, 2019). The findings from the vignette on gender norms were compared with the other gender role findings from the FGDs to observe consistency across themes. For each of the themes, illustrative quotes that most suitably captured them were selected.

## 3 RESULTS

**Figure 2:**
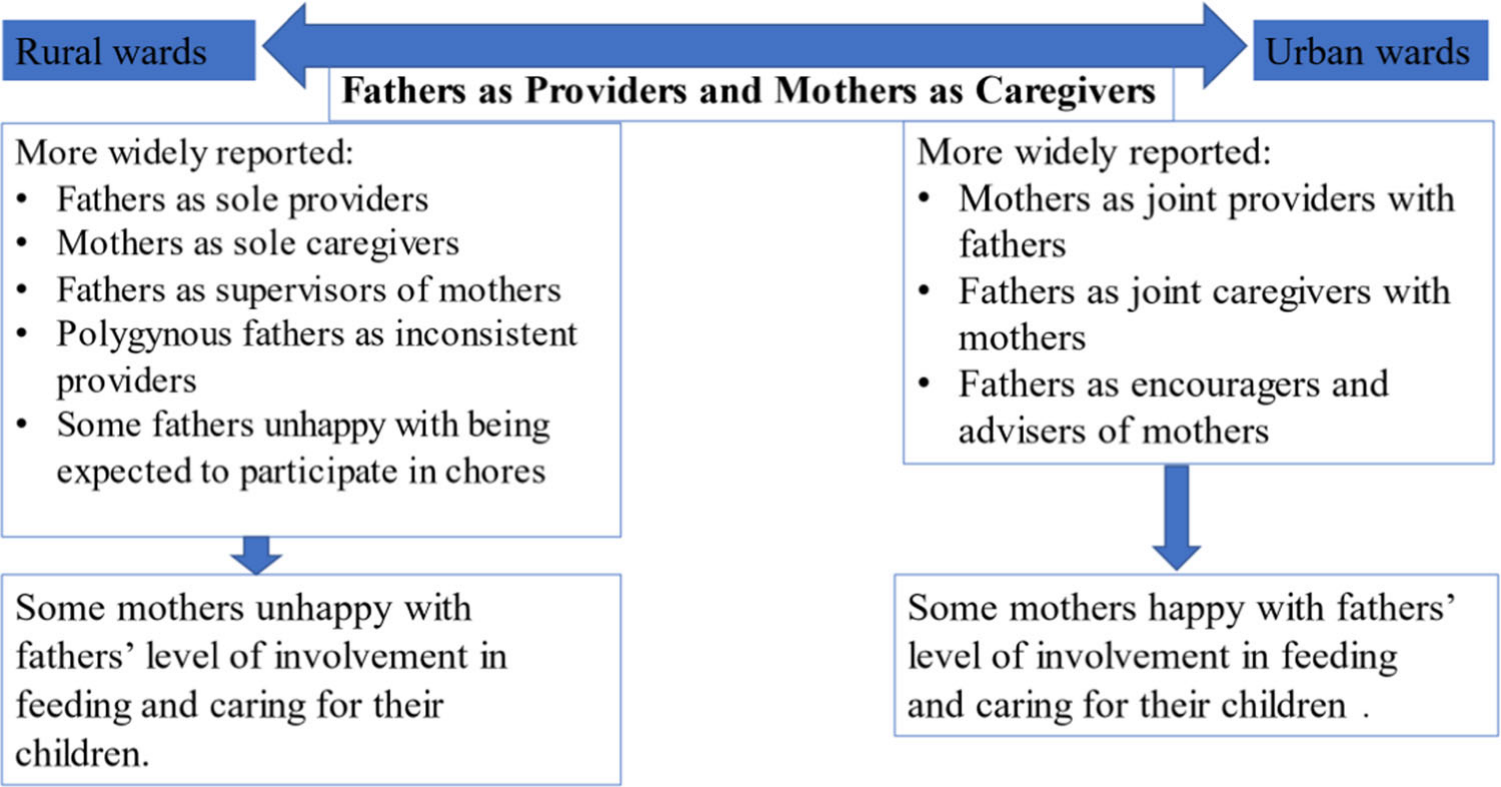
Household gender roles of mothers and fathers in the Alive & Thrive Kaduna study

The results are organized by the major themes identified in the FGDs including urban and rural differences in the roles of mothers and fathers. There were many similarities in themes across the FGDs for both mothers and fathers. The model in Figure 2, which represents a synthesis of these themes, depicts a continuum of household gender roles with the most salient roles being fathers as ‘providers’ and mothers as ‘caregivers’, which were common in both urban and rural locations. Other household gender roles were differentially reported in urban and rural wards, such as fathers as supervisors and encouragers of mothers. In addition, we contrast the injunctive and descriptive normative gender roles of mothers and fathers from the vignette included in the FGDs with fathers. Illustrative quotes are in Tables 1, 2, 3.

**Table 1:** Themes and illustrative quotes related to fathers and mothers as providers, caregivers, supervisors and encouragers

Emergent themes	Illustrative quotes
As sole caregivers, mothers are burdened with being responsible for feeding children diverse complementary foods	‘Now things have changed because of Covid‐19. Only the men doing business can be involved (in supporting child feeding). If it is a worker that has been laid off, he cannot be involved because he is suffering. He is also struggling to eat. How will he give to feed the child? You, the woman, the weight has been dropped on your shoulders and you do not have a choice’ Mothers FGD Igabi, rural ward.
As sole caregivers, household chores limit mothers' time availability to feed and care for their children	‘We all know that bringing up a young child puts a great strain on the mother particularly. I believe mothers need help in assisting them to do domestic work. Their difficulty is commitment in the area of kitchen work and taking care of the baby. There is a great demand on them’ Fathers FGD Rigachikun 1, urban ward.
As caregivers alongside mothers, fathers feed children with food mothers have prepared when mothers are involved with other household chores	‘Whatever my husband brings, we use it on the child, and we try to feed the child well. Now that I'm not at home, I am very sure the father will feed him. My child is always happy when my husband feeds him’ Mothers FGD Rigachikun 2, urban ward.
As supervisors of mothers, some fathers do not support mothers to practice recommended feeding behaviour	‘Sometimes men don't support when it comes to child feeding. A lot of discussion goes on because some men will tell you, you will not practice it and you must feed the child a certain way. You will see that what they say is not what you were told because it is not right. And when you see your friend or sister practicing it, you will want to do it too. Honestly, the men do not really encourage us very well sometimes’ Mothers FGD Turunku, rural ward.

**Table 2:** Themes and illustrative quotes related to injunctive and descriptive normative gender roles

Emergent themes	Illustrative quotes
*Injunctive normative gender roles*	
Fathers should be or are expected to be sole providers	‘The truth is that it is his duty as a man to fend for the family, so the issue of his wife helping him shouldn't arise. He should leave the house and work for the family’ Fathers FGD Rigachikun 2, urban ward.
Fathers should supervise mothers at home	‘It is expected that when the father provides all these foods, he should check the activities of the mother of the child to see if she is really playing her feeding role as it is expected of her. The feeding of the child with nutritious food is on the father. If he doesn't place some checks on the way the child is fed, then it will be a failure’ Fathers FGD Zangon Aya, rural ward.
Mothers should know how to combine household chores with caregiving	‘If the mother of a child does not have a timetable for her chores, then there will be a problem. As long as she has one, she will have time to feed her child the right food’ Fathers FGD Igabi, rural ward.
Mothers are expected to be better caregivers than fathers	‘Kabiru should advise his brother Yusuf to ask his wife, Laraba's mother to stop her business and face the task of taking care of the child. The child agrees more with the mother than the father’ Fathers FGD Kwarau, rural ward.
Fathers can marry other wives to assist mothers with household chores	‘Yusuf, the father, should marry another wife who will support Aisha with the household chores so that things will not be too much for her’ Fathers FGD Kwarau, rural ward.
*Descriptive normative gender roles*	
Fathers are providers and mothers are caregivers	‘Yusuf has gone out to look for food to bring back home while the mother takes care of the household chores. He has gone out to look for [food] so that he will get the type of foods that are important for the wellbeing of his wife and children’ Fathers FGD Rigasa 1, urban ward.
Mothers and fathers are joint providers	‘She is trying her best. Like it was said earlier, a woman can support her husband in providing [resources]. You see this is the situation here. She is trying with the small business she is doing to help provide [resources]. That is the reason she doesn't have time to care for her daughter’ Fathers FGD Turunku, rural ward.
Mothers and fathers are joint caregivers to their children	‘All that has been said is true. The responsibility of providing food is the father's because it is good for the father to go out and look for [food] to bring home, and make sure the wife prepares the food the way she is supposed to, in a timely manner. Stay there until she prepares it and feeds the children. If you see that they are not eating, play with them to make sure they eat. Because sometimes the mother will try to feed the child and the child will not eat. But when you, the father feeds the child, the child will eat’ Fathers FGD Kwarau, rural ward.
Fathers help with household chores	‘Sometimes the fathers can do the cooking themselves’ Fathers FGD Rigachikun 1, urban ward.

**Table 3:** Themes and illustrative quotes related to urban and rural differences

Emergent themes	Illustrative quotes
*Themes more salient in the rural wards*	
Rural mothers and fathers have distinct and separate roles	‘Mostly the male parents bring [food]and the female parents prepare it and feed the children’ Fathers FGD Turunku, rural ward.
Some rural fathers are less supportive	‘The way the men in the urban area care for their child, our husbands do not do the same. If anything comes up, it is the mother that deals with it’ Mothers FGD Igabi, rural ward.
Rural fathers have more wives, which keep them from adequately providing for their children	‘What we want is for them to help us women. Because it is not every man that can take responsibility. Our husbands here in the rural area have plenty wives and plenty children’ Mothers FGD Igabi, rural ward.
Rural mothers unhappy with rural fathers' involvement	‘I want to see changes in their involvement even if it is not much. They should provide for the children to eat’ Mothers FGD Kwarau, rural ward.
*Themes more salient in the urban wards*	
Urban mothers and fathers discuss and mutually agree on their roles	‘The first thing I ensure to do is to provide the necessary things that everyone in the house needs that will benefit them. Secondly, I spend time with my wife, the mother of the kids, discussing what needs to be done for each child, so that they will live a good and healthy life by feeding what they really need to eat. Thirdly, helping her with chores, I do not leave everything for her to do’ Fathers FGD Rigasa 2, urban ward.
Urban fathers help complete household chores in addition to providing the recommended foods	‘What I do as a father is that I often make myself available for my wife. Get her fruits because as a nursing mother she needs fruits. Most of the domestic work, we share together to make the burden less’ Fathers FGD, Rigachukun 1, urban ward.
Urban mothers are happy with urban fathers' involvement	‘Why I like my husband's involvement is because he takes it more seriously than I do. It pleases me because of the way my child is growing well’ Mothers FGD Rigasa 1, urban ward.

### 3.1 Fathers as ‘providers’ and mothers as ‘caregivers’

As ‘providers’, fathers are responsible for providing either money or food items to mothers. As ‘caregivers’, mothers are responsible for preparing the food, feeding the food to the children and carrying out other household chores: ‘The father provides the money; the mother buys the food and also prepares it for the child’ Mothers FGD Rigasa 2, urban ward.

Many fathers from both the urban and rural wards spoke about their roles as ‘providers’ not only the cultural expectation but also backed by the religious tenets of both Islam and Christianity. However, other fathers, who were mostly from the urban wards, spoke of instances when mothers support fathers as ‘joint‐providers’ or become ‘sole‐providers’ and explained that the demands of the current times do not allow fathers to be the only providers. Mothers also confirmed these roles and said this could occur when the father prioritizes his leisure activities over providing the food needed by children. It also happens when the father has no income, but the mother is involved in income‐generating activities. Some mothers mentioned the burden of being solely responsible for feeding their children the recommended foods.

Although mothers are caregivers to children in their homes, several mothers and fathers acknowledged that mothers' other household responsibilities, such as completing chores, can be a barrier to feeding children recommended foods. In view of that, some fathers said they help complete household chores and acknowledged that fathers helping with chores results in greater bonding within families and more respect from children: ‘When a father does all these [things], they [children] respect him. Children will obey their father because they know he is a responsible parent, and he will be an example to his children’ Fathers FGD Rigachukun 1, urban ward.

Some fathers encouraged other fathers to do similarly: ‘First of all, you the father are supposed to help out with chores. You know that women do not find it easy. By the time you are home, help your wife’ Fathers FGD Kwarau, rural ward. In the mothers' FGDs, some mothers confirmed that some fathers help with chores and contribute to food preparation. Other fathers also feed children with food mothers have prepared when mothers are involved with other household chores or when they are away from the home.

### 3.2 Fathers as ‘supervisors of mothers’

Some mothers also mentioned fathers in other supervisory roles, which they described as inhibiting mothers from feeding their children recommended foods. Some mothers indicated that fathers instruct them on ways to feed children that contradict recommendations they had received from the CHEWs. These mothers spoke about being discouraged by such circumstances.

### 3.3 Fathers as ‘encouragers/advisers'

Some fathers indicated that apart from ensuring the provision of the food needs of their children, they also provided encouragement and advice to mothers: ‘In all cases, love covers everything. You need to give them love and attention. So even if you provide all the food but love and attention are not there, the woman will not be encouraged as she is supposed to be. So, love and attention go a long way for the health and wellbeing of the child’ Fathers FGD Rigachikun 1, urban ward.

### 3.4 Injunctive and descriptive normative gender roles

Many of the findings from the vignette were consistent with the themes of fathers as providers and supervisors and mothers as caregivers. Fathers' discussions of ‘what Yusuf and Aisha do each day’ in comparison with suggestions about ‘what Yusuf should do’, revealed the descriptive and injunctive normative roles of mothers and fathers. Illustrative quotes are found in Table 2.

There were some fathers who expressed displeasure about Yusuf's inability to provide for his family using phrases such as ‘man up and fend for his family’ and ‘incompetent as the man of the house’ Fathers FGD Rigachikun 1, urban ward. Other fathers expressed displeasure about mothers' supporting fathers as providers and joked that as mothers were taking over fathers' roles, fathers should also take over mothers' roles as caregivers: ‘Since it has happened like that and the sky is now on the ground, the mother has taken the father's job in providing food. Since he doesn't have [a job], he should take the mother's place. All that she does to care for and feed the child food, the father should do it’ Fathers FGD Igabi, rural ward. Some fathers also spoke of fathers' normative responsibility of supervising mothers in the home to ensure mothers are performing their caregiver roles well.

In relation to mothers' caregiving responsibilities, fathers spoke of the expectation that mothers combine household chores with child caregiving in a manner that allows both to be completed adequately. Some fathers went on further to request that mothers' focus on their caregiving responsibilities because fathers do not have the ability to take care of children. Other fathers proposed that domestic helpers, other family members or other wives (polygynous households) could help mothers with household chores rather than fathers. These perspectives on what Yusuf ‘should do’ created a picture of the injunctive normative gender roles of fathers as sole providers and supervisors and mothers as sole caregivers without support from either of them in fulfilling those responsibilities.

On the other hand, some fathers, on describing what Yusuf and Aisha do each day, acknowledged that sometimes mothers and fathers play roles that do not align with the socioculturally expected behaviours. These fathers mentioned mothers and fathers jointly providing resources and supporting caregiving as examples of the roles mothers and fathers are playing. The fathers went on to admit that they also helped with household chores from time to time in their own homes.

### 3.5 Urban and rural differences in household roles of mothers and fathers

There were themes that were more salient in the FGDs of the mothers and fathers from rural wards compared with urban wards (Figure 2). The details are reported below with the key themes and illustrative quotes in Table 3. Many fathers from the rural wards communicated their displeasure with fathers being expected to perform household chores in addition to providing for their households. Most mothers and fathers from rural wards also communicated very distinct and separate roles for mothers and fathers in the feeding and care of children. Some fathers stated that fathers are solely providers and supervisors and mothers are solely caregivers. In some cases, some mothers from the rural wards acknowledged that fathers are less helpful and supportive in caring for their children compared with fathers from urban areas. There were also some mothers who expressed their desire that fathers put more effort into providing diverse foods. Furthermore, although they were few, there were also some rural mothers who mentioned rural fathers' responsibilities towards multiple children with other wives (polygynous homes) as keeping them from being able to provide adequately for their children.

In contrast, mothers and fathers from urban wards communicated a less traditional approach to their roles in ensuring children are fed diverse complementary foods. Both mothers and fathers explained that their roles were based on mutual agreement rather than on defined traditional normative responsibilities. Some fathers spoke about consultative discussions and decision‐making processes with mothers about what needed to be done at home for their children. Other urban fathers also spoke about more hands‐on approaches including participation in household tasks in addition to providing the recommended foods for not only their children but also for mothers as well. Another difference was that, unlike the rural wards, there were mothers who spoke positively about what fathers were doing to support feeding their children diverse complementary foods. These mothers indicated that the fathers were enthusiastic about being involved in feeding and caring for their children.

## 4 DISCUSSION

This study sought to identify and thematically describe how household gender roles influence feeding children diverse foods after the implementation of a programme that engaged fathers to support complementary feeding of their young children. Our findings revealed that the most salient theme in the FGDs was the father as ‘provider’ and mother as ‘caregiver’. This indicates that mothers and fathers hold traditional attitudes about their roles in feeding children diverse complementary foods, but with some differences when comparing urban and rural wards. Traditional gender roles norms are often prescriptive about mothers and fathers' roles in the home, particularly as they relate to child feeding and care (Dickin et al., 2021; Eagly & Wood, 2012) and in this context were more widely reported in the rural compared with the urban wards.

There were some themes from the fathers FGDs about the roles of fathers limited to the provision of resources for their households and supervising mothers with limited involvement in the ‘hands‐on’ activities that support complementary feeding. This is in line with social role theory and consistent with previous research findings in Nigeria, Malawi, Kenya and South Africa, which show that mothers continue to be defined by their caregiving roles, while fathers are seen as providers and decision makers with respect to complementary feeding (Bezner Kerr, 2005, 2016; Eagly & Wood, 2012; Ejuu, 2016; Ene‐Obong et al., 2017; Erzse et al., 2021; Kram et al., 2016; Lamb, 2000; Rakotomanana et al., 2021; Thuita et al., 2015).

Other dimensions of mothers' injunctive normative caregiving roles and responsibilities were observed when fathers revealed their expectation that mothers be more organized with their time so they can adequately perform their caregiving roles without fathers' support. Previous studies have shown that mothers are burdened by many responsibilities, which often limits the amount of time they have for child feeding and care (Hackett et al., 2015; Komatsu et al., 2018; Nankumbi & Muliira, 2015; Popkin, 1980).

This study also found that mothers and fathers in some households are performing other ‘non‐traditional’ gender normative roles, such as mothers contributing to the provision of food/resources and fathers supporting the responsibilities of food preparation, child feeding and household chores, despite social disapproval. Mothers as economic contributors can lead to more equitable household gendered decision making (Kadiyala et al., 2014), more equitable intrahousehold food allocation (Harris‐Fry et al., 2017), improved availability of nutritious foods for young children (Harris‐Fry et al., 2020) and improved complementary feeding practices (Bezner Kerr, 2005, 2016; Chintalapudi et al., 2018; Kuche et al., 2020; Oddo & Ickes, 2018; Sariyev et al., 2020).

Although ‘non‐traditional’ gender normative roles were discussed in the FGDs, some rural fathers joked about them because such roles are contrary to the injunctive gender norms and roles of mothers and fathers. These ‘shifts’ from the ‘traditional’ household roles were more salient among urban than rural fathers. In the urban wards, mothers and fathers reported using dialogue and mutual agreement strategies to decide tasks they should perform, while mothers and fathers from rural wards described fathers as providers and supervisors and mothers as caregivers. Furthermore, mothers from urban wards often reported the fathers were helpful and enthusiastic, while fathers from rural wards reported their unwillingness and displeasure about being expected to participate in household chores. Similar to findings from a previous study on male roles and household chores in Nigeria (Akanle et al., 2017), these findings suggest that urbanization may contribute to the more positive attitudes of urban fathers towards caregiving compared with rural fathers. These descriptions also suggest the need for strategic targeting of rural communities in Kaduna State and Nigeria as a whole using gender transformative approaches to confront and transform the gender‐related social norms related to mothers' and fathers' roles in child feeding and care.

Although other studies have reported that fathers can play other roles in the home to support complementary feeding by contributing to domestic and caregiving work (Doyle et al., 2018; Lamb, 2000; Martin et al., 2021), this was more salient among fathers in the urban wards than the rural wards. It is worth noting that as this was not a gender‐transformative intervention, fathers were not encouraged to be more involved in domestic and caregiving work. Despite this, mothers and fathers who reported fathers who help more with domestic and caregiving work reported positive effects such as stronger relationships within families, more respect for fathers and positive home environments. Given the suggested positive effects of fathers' participation in domestic and caregiving work in their homes, there is the need for gender‐transformative programming specific to the Kaduna context. Such gender‐transformative programmes could focus on leveraging some of the benefits reported in this study as motivations for more fathers to recognize and be more intentional about their joint caregiving responsibilities with mothers for sustained impact (Bezner Kerr, 2016; Doyle et al., 2014; Ejuu, 2016; Lamb, 2000; Martin et al., 2020; Mawusi, 2013).

This study also found that some fathers recognized their roles to emotionally support mothers as an important part of their involvement in complementary feeding. This finding is consistent with the emotional and psychological support component of father involvement identified by Lamb (2000). On the other hand, despite these positive sentiments, some mothers and fathers reported fathers assumed dominating roles such as ‘supervisors’ of mothers' actions to ensure mothers were adequately feeding and caring for children, which other studies have also reported (Martin et al., 2020). In some instances, fathers in these supervisory roles provided advice that conflicted with complementary feeding recommendations made by CHEWs. In addition to addressing possible negative influences of misinformed fathers, future intervention research should also focus on exploring more positive aspects of masculine norms within the African context to be able to emphasize them in complementary feeding SBCC messages for fathers.

### 4.1 Strengths and limitations

Our study has several strengths, including the collection of data from both mothers and fathers about fathers' involvement in child feeding and care, use of the vignette in the father FGDs to explore fathers' perspectives on household gender normative roles without judgement and use of repeated layers of coding and analysis to highlight the urban and rural differences in household gender roles.

This study also has some limitations, including non‐inclusion of the vignette in the mothers' FGD guide, fathers' self‐reports about the positive effects of their involvement in complementary feeding in the results and the potential for lack of data saturation. While the vignette allowed us to observe a wide range of attitudes of fathers towards normative gender roles in child feeding and care, we were unable to similarly explore mothers' perspectives. Future research studying how household gender roles influence complementary feeding should include similar vignettes in FGDs for both mothers and fathers. While fathers may not have accurately described child feeding, family relationships and home environments, similar themes were noted in the mother focus group discussions, which made fathers' self‐report about the positive effects of their involvement in complementary feeding less of a concern. Finally, our study's sampling approach took into consideration the diverse setting of Kaduna State (urban or rural, ethnic, social and religious diversity); hence, a total of 16 focus groups were planned and conducted. While the potential for lack of data saturation is a concern for qualitative studies, the FGD participant responses in early and later FGDs were similar, indicating that we likely achieved saturation of results in later FGDs for our study.

## 5 CONCLUSIONS

Our research showed that the traditional roles of fathers as providers and mothers as caregivers, which are guided by injunctive gender norms, are common in Kaduna State. Despite this, injunctive and descriptive normative roles sometimes differed, such as when mothers were sole providers or joint providers, or fathers played caregiving roles in their homes. More often in urban wards compared with rural wards, mothers and fathers discussed and agreed on their roles at home with mothers and fathers jointly providing and caring for their children. In rural wards, fathers were sole providers and supervisors of mothers and mothers were sole caregivers. Our research findings point to the need for more context‐specific approaches that address prevalent gender normative roles in complementary feeding in a variety of settings.

## CONFLICT OF INTERESTS

SK and SB are employed by Alive & Thrive. They were involved in the implementation and evaluation of the intervention in Nigeria, but not in data collection or analysis for this study. All other authors report no conflict of interests.

## AUTHOR CONTRIBUTIONS

SLM, DA and VLF conceptualized this study. SLM, VLF, SK and SB contributed to study design and implementation. AI collected the data. DA, SLM, BW, KK, VLF and MEB contributed to the analysis. DA drafted the paper. All authors contributed to critically revising and approving the final version of this manuscript.

## Data Availability

The data that support the findings of this study are available from the corresponding author upon request after receiving approval from Alive & Thrive.
